# Clinical spectrum and prognostic impact of cancer in critically ill patients with HIV: a multicentre cohort study

**DOI:** 10.1186/s13613-023-01171-4

**Published:** 2023-08-22

**Authors:** Piotr Szychowiak, Thierry Boulain, Jean-François Timsit, Alexandre Elabbadi, Laurent Argaud, Stephan Ehrmann, Nahema Issa, Emmanuel Canet, Frédéric Martino, Fabrice Bruneel, Jean-Pierre Quenot, Florent Wallet, Élie Azoulay, François Barbier

**Affiliations:** 1 Médecine Intensive Réanimation, Centre Hospitalier Régional d’Orléans, 14, Avenue de L’Hôpital, 45100 Orléans, France; 2 Réanimation Médicale et des Maladies Infectieuses, Centre Hospitalier Universitaire Bichat-Claude Bernard, Assistance Publique-Hôpitaux de Paris, Paris, France; 3 Médecine Intensive Réanimation, Centre Hospitalier Universitaire Tenon, Assistance Publique-Hôpitaux de Paris, Paris, France; 4 Médecine Intensive Réanimation, Centre Hospitalier Universitaire Edouard Herriot, Hospices Civils de Lyon, Lyon, France; 5 Médecine Intensive Réanimation, Centre Hospitalier Universitaire de Tours, Tours, France; 6 Médecine Intensive Réanimation, Centre Hospitalier Universitaire de Bordeaux, Bordeaux, France; 7 Médecine Intensive Réanimation, Centre Hospitalier Universitaire de Nantes, Nantes, France; 8 Médecine Intensive Réanimation, Centre Hospitalier Universitaire de La Guadeloupe, Pointe-À-Pitre, France; 9 Réanimation et Unité de Surveillance Continue, Centre Hospitalier de Versailles, Le Chesnay, France; 10 Médecine Intensive Réanimation, Centre Hospitalier Universitaire de Dijon-Bourgogne, Dijon, France; 11 Médecine Intensive Réanimation, Centre Hospitalier Universitaire Lyon Sud, Hospices Civils de Lyon, Lyon, France; 12 Médecine Intensive Réanimation, Centre Hospitalier Universitaire Saint-Louis, Assistance Publique-Hôpitaux de Paris, Paris, France

**Keywords:** Human immunodeficiency virus, Cancer, Intensive care unit, Treatment limitation decision, Outcome

## Abstract

**Background:**

Both AIDS-defining and non-AIDS-defining cancers (ADC/NADC) predispose people living with HIV (PLHIV) to critical illnesses. The objective of this multicentre study was to investigate the prognostic impact of ADC and NADC in PLHIV admitted to the intensive care unit (ICU).

**Methods:**

All PLHIV admitted over the 2015–2020 period in 12 university-affiliated ICUs in France were included in the study cohort. The effect of ADC and NADC on in-hospital mortality (primary study endpoint) was measured through logistic regression with augmented backward elimination of potential independent variables. The association between ADC/NADC and treatment limitation decision (TLD) during the ICU stay (secondary study endpoint) was analysed. One-year mortality in patients discharged alive from the index hospital admission (exploratory study endpoint) was compared between those with ADC, NADC or no cancer.

**Results:**

Amongst the 939 included PLHIV (median age, 52 [43–59] years; combination antiretroviral therapy, 74.4%), 97 (10.3%) and 106 (11.3%) presented with an active NADC (mostly lung and intestinal neoplasms) and an active ADC (predominantly AIDS-defining non-Hodgkin lymphoma), respectively. Inaugural admissions were common. Bacterial sepsis and non-infectious neoplasm-related complications accounted for most of admissions in these subgroups. Hospital mortality was 12.4% in patients without cancer, 30.2% in ADC patients and 45.4% in NADC patients (*P* < 0.0001). NADC (adjusted odds ratio [aOR], 7.00; 95% confidence interval [CI], 4.07–12.05) and ADC (aOR, 3.11; 95% CI 1.76–5.51) were independently associated with in-hospital death after adjustment on severity and frailty markers. The prevalence of TLD was 8.0% in patients without cancer, 17.9% in ADC patients and 33.0% in NADC patients (*P* < 0.0001)—organ failures and non-neoplastic comorbidities were less often considered in patients with cancer. One-year mortality in survivors of the index hospital admission was 7.8% in patients without cancer, 17.0% in ADC patients and 33.3% in NADC patients (*P* < 0.0001).

**Conclusions:**

NADC and ADC are equally prevalent, stand as a leading argument for TLD, and strongly predict in-hospital death in the current population of PLHIV requiring ICU admission.

**Supplementary Information:**

The online version contains supplementary material available at 10.1186/s13613-023-01171-4.

## Background

The epidemiology of cancer in people living with HIV (PLHIV) has markedly evolved over the past decades owing to augmented access to combination antiretroviral therapy (cART). The incidence and attributable mortality of AIDS-defining cancers (ADC) (namely, high-grade B-cell non-Hodgkin lymphoma [NHL], Kaposi sarcoma, and carcinoma of the cervix) have globally declined though PLHIV remain at higher hazard for these neoplasms than seronegative individuals, especially in case of undiagnosed HIV infection or social issues that jeopardise sustainable cART adherence [1–4]. Conversely, extended life expectancy, lifestyle risk factors, certain viral co-infections and chronic systemic inflammation ensuing from silent HIV replication may all contribute to the growing prevalence of non-AIDS-defining cancers (NADC) which nowadays prevail and stand as a leading cause of death in the overall population of cART-treated PLHIV [5–8].

Both ADC and NADC predispose PLHIV to life-threatening events resulting from sepsis, metabolic disorders, mechanical complications, or chemotherapy-related toxicity [9, 10]. Yet, granular data are lacking to appraise the current impact of cancers on the clinical features and outcomes of critical illnesses in these patients [11]. Next, palliative approaches now constitute an essential component of care for PLHIV ageing with severe comorbidities and altered performance status [12]; however, treatment limitation decisions (TLD) remain under-investigated in those admitted to the intensive care unit (ICU) and how cancers affect TLD in this setting is unknown [11]. These elements appear pivotal to improve the collaborative management of PLHIV by intensivists, infectious diseases specialists and oncologists, define the appropriate level of care, and help rationalising the utilisation of ICU resources in this changing patient population.

The primary objective of this multicentre cohort study was to measure the effect of NADC and ADC on hospital mortality in a contemporary population of PLHIV admitted to the ICU. The secondary objective was to appraise the impact of cancer on TLD in these patients. The association between cancer status and one-year mortality in patients alive at hospital discharge was investigated as an exploratory objective.

## Patients and methods

### Study design and patient inclusion

This study was conducted in 12 medical ICUs located in university-affiliated hospitals in France and contributing to the GRRROH (*Groupe de Recherche Respiratoire en Réanimation Onco-Hématologique*, www.grrroh.fr) and CRICS-TRIGGERSEP (Clinical Research in Intensive Care and Sepsis—Trial Group for Global Evaluation and Research in Sepsis, www.crics-triggersep.org) networks for research in intensive care (see the Additional file 1 for details on centre recruitment and characteristics). All adult PLHIV admitted to these ICUs over a 5.5 year period (from January 2015 to June 2020) were retrospectively identified by local investigators through hospital coding databases using the International Classification of Diseases–10th revision items related to HIV infection (that is B20-24, Z21, and R75) and included in the study cohort provided that there was no missing information regarding cART use at ICU admission and the vital status at hospital discharge. Only the first ICU stay was analysed in patients with multiple admissions over the inclusion period. The study protocol was approved by the ethical committee of the French Intensive Care Society on August 3, 2020 (report n°CE-SRLF-20–70); the requirement for informed consent was waived owing to the observational design. Results are reported according to the STROBE guidelines [13].

### Data collection and definitions

The patient medical records from the ICU and the downstream unit were anonymised by local investigators and centralised before data extraction with a standardised form. Vital status at last follow-up visit in the participating centre was also collected, when available.

cART was defined according to the current International Antiviral Society guidelines [14]. When available, CD4 cell count and HIV viral load were collected within the 6 months preceding ICU admission for patients with previously known HIV infection and at ICU admission for those with newly diagnosed seropositivity. Missing values are indicated in Additional file 1: Table S1.

AIDS-defining conditions were defined according to the Centers for Diseases Control and Prevention classification, with primary brain lymphoma, Burkitt lymphoma, other high-grade B-cell NHL, Kaposi sarcoma and cervix cancer being identified as ADC [15]. Other solid and haematological neoplasms were classified as NADC. NADC and ADC were considered active in patients with either inaugural admission, response to first-line therapy, relapse, or refractory disease. Neoplasms with proven remission after first-line therapy were classified as inactive.

The primary diagnoses of the ICU stay were ventilated as cancer-related non-infectious complications, non-infectious complications of chronic conditions other than cancer, bacterial sepsis, non-AIDS-defining non-bacterial infections, AIDS-defining opportunistic infections, cART-related toxicity, and others.

The primary study endpoint was in-hospital mortality. The secondary study endpoint was the existence of a TLD (either withholding or withdrawal of organ support) during the ICU stay, as stated in the medical record file. Written arguments for TLD were collected. One-year mortality in patients discharged alive from the index hospital admission was investigated as an exploratory endpoint.

## Statistical analyses

Data are expressed as median (interquartile range) for continuous variables and number (percentage) for categorical variables, unless otherwise indicated. The analysis of variance test and the Kruskal–Wallis test were used to compare continuous variables with normal and non-normal distributions, respectively. The Fisher’s exact test or the *χ*^2^ test, as appropriate, was used to compare categorical variables.

To assess the patient characteristics that could have influenced the occurrence of the primary study endpoint, we used a logistic regression model with in-hospital death as the dependent variable. As independent variables, we first entered in a global starting model a number of patient characteristics that could have been linked to this endpoint. To reduce the number of potential independent variables per event, we then proceeded to augmented backward elimination (ABE) that combines the standardised change-in-estimate criterion with significance-based backward elimination, with liberal criteria to keep independent variables in the model (threshold values, *P* < 0.35 and change-in-estimate < 35%) as to minimise the risk of eliminating important explanatory variables [16, 17]. Passive variables that were considered systematically associated with in-hospital death (namely age, sex, and the SOFA score value at ICU admission) were kept in the models. Other active variables that could have been linked to in-hospital mortality or modified the influence of passive variables were also introduced in the starting model submitted to ABE. This procedure was repeated on 1000 bootstrap samples (with replacement) of the study population. Potentially explanatory variables (exposed with their adjusted odd ratio [aOR] and 95% confidence interval [CI]) were retained in the final model if they were selected in more than 50% of the bootstrap samples, with a root mean square difference ratio < 1.5 and an absolute relative conditional bias of less than 50%. Discriminative ability of the model was assessed through area under the receiver operating characteristics curve (AUROC).

The cumulative survival at one year in patients discharged alive from the hospital was compared between the three groups (active ADC, active NADC, and no active cancer) through Kaplan–Meier analyses and the log-rank test, with right-censoring at the date of last follow-up information.

All analyses were conducted using the R software version 3.5.1 (http://www.R-project.org). Two-tailed *P* values < 0.05 were considered statistically significant.

## Results

### Baseline characteristics of the study population

A total of 939 PLHIV were included (Table 1 and Additional file 1: Fig. S1, additional characteristics of the study population in Additional file 1: Table S1). Amongst them, 812 (86.5%) were known as HIV-infected prior to hospital admission (time from HIV diagnosis, 13.6 [2.8–21.2] years; CD4 cell count, 370 [180–600] per µL; HIV viral load, < 50 [< 50 – < 50] copies per µL), including 699 (74.4%) receiving cART. The diagnosis of HIV infection was made during the index hospitalisation for the remaining 127 patients (13.5%) (CD4 cell count, 51 [20–147] cells per µL; HIV viral load, 5.10^5^ [10^5^–10^6^] copies per µL). Chronic obstructive pulmonary disease, coronary artery disease, liver cirrhosis and renal conditions were the most common comorbidities (Table 1). Twenty-four patients (2.6%) were solid organ transplant recipients, including 21 kidney transplant recipients. Of note, the 5.5-year inclusion period ended in June 2020, that is barely 3 months after the beginning of the first COVID-19 wave in France, with only 7 patients (0.7%) admitted for COVID-19-related respiratory failure.

**Table 1:** Characteristics of the study population according to the cancer status

	All patients (*n* = 939)	Patients without active cancer (*n* = 736)	Patients with active ADC (*n* = 106)	Patients with active NADC (*n* = 97)	*P-*value
Male sex	670 (71.3)	514 (69.8)	79 (74.5)	77 (79.4)	0.11
Age, years	52 (43–59)	52 (43–59)	50 (42–57)	55 (47–62)	0.02
Precarity^a^	136 (14.5)	104 (14.1)	16 (15.1)	16 (16.5)	0.81
WHO performance status					
0	514 (54.7)	454 (61.7)	41 (38.7)	19 (19.6)	< 0.0001
1–2	334 (35.6)	224 (30.4)	49 (46.2)	61 (62.9)	
3–4	91 (9.7)	58 (7.9)	16 (15.1)	17 (17.5)	
HIV-related characteristics					
Newly diagnosed HIV infection^b^	127 (13.5)	105 (14.3)	18 (17.0)	4 (4.1)	0.01
CD4 cell count at admission, per µL	51 (20–147)	50 (20–150)	57 (38–94)	360 (340–380)	< 0.0001
Previously known HIV infection	812 (86.5)	631 (85.7)	88 (83.0)	93 (95.9)	0.01
cART at admission	699 (74.4)	539 (73.2)	72 (67.9)	88 (90.7)	< 0.0001
Baseline CD4 cell count, per µL^c^	370 (180–600)	436 (230–682)	221 (95–484)	301 (170–470)	< 0.0001
History of AIDS-defining OI^d^	301 (32.1)	231 (31.4)	37 (34.9)	33 (34.0)	0.96
History of HIV encephalitis^d^	21 (2.2)	16 (2.2)	5 (4.7)	0	0.07
History of Castleman disease^d^	16 (1.7)	12 (1.6)	1 (0.9)	0	0.40
History of ADC (remission or cured)^d^					
Any	50 (5.3)	41 (5.6)	0	9 (9.3)	0.01
AIDS-defining NHL	11 (1.2)	9 (1.2)	0	2 (2.1)	0.38
Kaposi sarcoma	39 (4.2)	31 (4.2)	0	8 (8.2)	0.01
Cervix cancer	1 (0.1)	1 (0.1)	0	0	NA
History of NADC (remission or cured)^d^					
Any	53 (5.6)	51 (6.9)	2 (1.9)	0	0.004
Solid NADC	41 (5.5)	39 (5.3)	2 (1.9)	0	0.02
Haematological NADC	13 (1.4)	13 (1.8)	0	0	0.16
Chronic conditions					
Respiratory	199 (21.2)	172 (23.4)	7 (6.6)	20 (20.6)	0.0004
COPD	107 (11.4)	88 (12.0)	2 (1.9)	17 (17.5)	0.001
Cardiac	175 (18.6)	149 (20.2)	12 (11.3)	14 (14.4)	0.05
Coronary heart disease	92 (9.8)	80 (10.9)	3 (2.8)	9 (9.3)	0.03
Renal	171 (18.2)	153 (20.8)	6 (5.7)	12 (12.4)	0.0002
Hepatic	200 (21.3)	168 (22.8)	10 (17.9)	22 (22.7)	0.008
Chronic HBV infection	72 (7.7)	56 (7.6)	6 (5.7)	10 (10.3)	0.46
Chronic HCV infection	111 (11.8)	93 (12.6)	5 (4.7)	13 (13.4)	0.06
Liver cirrhosis	74 (7.9)	58 (7.9)	4 (3.8)	12 (12.4)	0.08
Neurological	105 (11.2)	90 (12.2)	3 (2.8)	12 (12.4)	0.01
Psychiatric	114 (12.1)	101 (13.7)	8 (7.5)	5 (5.2)	0.02
Solid organ transplantation	24 (2.6)	24 (3.3)	0	0	0.03
Mode of ICU admission					
Direct from the emergency department	603 (64.2)	525 (71.3)	33 (31.1)	45 (46.4)	< 0.0001
Transfer from wards	336 (35.8)	211 (28.7)	73 (68.9)	52 (53.6)	
Time from hospital admission, days	7 (3–18)	6 (3–17)	9 (4–18)	9 (3–17)	< 0.0001
SAPS 2 at ICU admission	36 (26–51)	34 (23–47)	51 (39–63)	41 (30–56)	< 0.0001
SOFA score at ICU admission	4 (2–6)	4 (2–6)	4 (2–8)	4 (2–7)	0.06
Type of ICU admission					
Medical	904 (96.3)	710 (96.5)	105 (99.1)	89 (91.8)	0.009
Unscheduled surgery	13 (1.4)	12 (1.6)	0	1 (1.0)	
Scheduled surgery	22 (2.3)	14 (1.9)	1 (0.9)	7 (7.2)	
Main reason for ICU admission					
Acute respiratory failure	320 (34.1)	262 (35.6)	27 (25.5)	31 (32.0)	< 0.0001
Sepsis/septic shock	172 (18.3)	127 (17.3)	23 (21.7)	22 (22.7)	
Coma^e^	165 (17.6)	139 (18.9)	14 (13.2)	12 (12.4)	
Acute kidney failure	56 (6.0)	43 (5.8)	8 (7.5)	5 (5.2)	
Drug overdose	47 (5.0)	46 (6.3)	0	1 (1.0)	
Metabolic	47 (5.0)	17 (2.3)	21 (19.8)	9 (9.3)	
Shock (other than septic)	28 (3.0)	24 (3.3)	1 (0.9)	3 (3.1)	
Cardiac arrest	16 (1.7)	14 (1.9)	0	2 (2.1)	
Others	88 (9.3)	64 (8.7)	12 (11.3)	12 (12.4)	
Neutropenia at ICU admission	46 (4.9)	18 (2.4)	19 (17.9)	9 (9.3)	< 0.0001
Main diagnosis of the ICU stay					
Cancer-related non-infectious complication	116 (12.4)	–	69 (65.1)	47 (48.5)	NA
NADC	47 (5.0)	–	–	47 (48.5)	NA
ADC	69 (7.7)	–	69 (65.1)	–	NA
Tumor lysis syndrome	27 (3.1)	–	27 (25.5)	–	NA
Non-infectious complication of chronic condition^f^	242 (25.8)	232 (31.5)	1 (0.9)	9 (9.3)	< 0.0001
Respiratory	46 (4.9)	44 (6.0)	0	2 (2.1)	< 0.0001
Cardiac	41 (4.4)	39 (5.3)	1 (0.9)	1 (1.0)	
Renal	28 (3.0)	28 (3.8)	0	0	
Hepatic	12 (1.3)	9 (1.2)	0	3 (3.1)	
Neurological	24 (2.6)	23 (3.1)	0	1 (1.0)	
Psychiatric/addiction	65 (6.9)	63 (8.6)	0	2 (2.1)	
Others	26 (2.8)	26 (3.5)	0	0	
Bacterial sepsis^g^	263 (28.0)	206 (28.0)	25 (23.6)	32 (33.0)	0.33
Non-AIDS-defining non-bacterial infection^h^	43 (4.6)	42 (5.7)	1 (0.9)	0	0.007
AIDS-defining OI	156 (16.6)	147 (20.0)	7 (6.6)	2 (2.1)	< 0.0001
*Pneumocystis jirovecii* pneumonia	69 (7.3)	66 (9.0)	3 (2.8)	0	0.001
Cerebral toxoplasmosis	30 (3.2)	28 (3.8)	2 (1.9)	0	0.1
Tuberculosis	14 (1.5)	12 (1.6)	1 (0.9)	1 (1.0)	0.79
Other OI	43 (4.6)	41 (5.6)	1 (0.9)	1 (1.0)	0.02
cART-related toxicity	7 (0.7)	7 (1.0)	0	0	0.38
Miscellaneous	111 (11.9)	102 (13.9)	3 (2.8)	9 (9.3)	0.003
Organ support in the ICU					
High-flow nasal oxygen therapy	96 (10.2)	73 (9.9)	9 (8.5)	14 (14.4)	0.32
Non-invasive ventilation	73 (7.8)	63 (8.6)	2 (1.9)	8 (8.2)	0.06
Invasive mechanical ventilation	301 (32.1)	228 (31.0)	37 (34.9)	36 (37.1)	0.38
Vasopressors	242 (25.8)	169 (23.0)	42 (39.6)	31 (32.0)	0.0004
Renal replacement therapy for AKI	105 (11.2)	73 (9.9)	24 (22.6)	8 (8.2)	0.0002
VA-ECMO	4 (0.4)	4 (0.5)	0	0	0.57
VV-ECMO	10 (1.1)	9 (1.2)	0	1 (1.0)	0.52
Chemotherapy in the ICU	71 (7.6)	8 (1.1) ^i^	50 (47.2)	13 (13.4)	< 0.0001
TLD during the ICU stay	110 (11.7)	59 (8.0)	19 (17.9)	32 (33.0)	< 0.0001
Organ support withdrawal	38 (4.0)	22 (3.0)	4 (3.8)	12 (12.4)	< 0.0001
Organ support withholding	72 (7.7)	37 (5.0)	15 (14.2)	20 (20.6)	< 0.0001
Outcomes					
ICU length of stay, days	5 (3–9)	4 (3–9)	6 (3–11)	5 (3–9)	0.03
Hospital length of stay, days	19 (10–36)	17 (9–33)	33 (17–60)	22 (11–36)	< 0.0001
ICU readmission in ICU survivors	48/827 (5.8)	36/667 (5.4)	10/87 (11.5)	2/73 (2.7)	0.04
In-ICU death	112 (11.9)	69 (9.4)	19 (17.9)	24 (24.7)	< 0.0001
In-hospital death, overall	167 (17.8)	91 (12.4)	32 (30.2)	44 (45.4)	< 0.0001
Post-ICU death, overall	55 (5.9)	22 (3.0)	13 (12.3)	20 (20.6)	< 0.0001
Post-ICU death related to cancer	24 (2.6)	–	8 (7.5)	16 (16.5)	NA

Data are exposed as number (percentage) or median (interquartile range)

ADC, AIDS-defining cancer; NADC, non-AIDS-defining cancer; WHO, World Health Organization; cART, combination antiretroviral therapy; AIDS, acquired immune deficiency syndrome; OI, opportunistic infection; PML, progressive multifocal leukoencephalopathy; ADC, AIDS-defining cancer; NHL, non-Hodgkin lymphoma; COPD, chronic obstructive pulmonary disease; ICU, intensive care unit; SAPS 2, simplified acute physiology score 2; SOFA, sepsis-related organ failure assessment; ICU, intensive care unit; AKI, acute kidney injury; VA/VV-ECMO, veno-arterial/veno-venous extracorporeal membrane oxygenation; TLD, treatment limitation decision

^a^Homeless (*n* = 29), migrants (*n* = 54), incarcerated patient (*n* = 1) and other complex social situations (*n* = 52);

^b^Diagnosis of HIV infection during the same hospital stay (inaugural admission);

^c^Within 6 months prior to ICU admission;

^d^Not active at ICU admission (remission or cured for neoplasms);

^e^Excluding patients with drug overdose;

^f^Non-neoplastic chronic conditions;

^g^See Additional file 1: Table S2 for details;

^h^Malaria (*n* = 20), COVID-19 (*n* = 7), influenza (*n* = 7), others (*n* = 9);

^i^Chemotherapy for Castleman disease (*n* = 2) and/or haemophagocytic lymphohistiocytosis (*n* = 6)

### Prevalence and spectrum of ADC and NADC

Overall, 106 (11.3%) and 97 (10.3%) patients presented with an active ADC and an active NADC, respectively. Solid cancers—mostly lung and intestinal neoplasms—accounted for 62 (63.9%) of NADC, with a metastatic stage in half of cases (Table 2). Hodgkin disease, non-AIDS-defining NHL and acute leukaemia were equally distributed amongst haematological NADC. Burkitt lymphoma and other AIDS-defining NHL represented 86 (81.1%) of ADC whilst only 19 cases of Kaposi sarcoma were identified (Table 2). The diagnosis of neoplasm was made during the index hospitalisation in 30 (48.3%), 21 (60.0%) and 65 (75.6%) of patients with solid NADC, haematological NADC and haematological ADC, respectively (*P* = 0.005). HIV infection and cancer were co-diagnosed during the index hospitalisation in 18 (17.0%) patients with ADC. Patients with NADC were older, received cART more frequently, had more non-neoplastic chronic conditions and presented with a worst performance status than those with ADC (Table 1). None of the solid organ transplant recipients had an active cancer at ICU admission.

**Table 2:** Features of active cancers and association with in-hospital mortality in the study population

Type and stage of cancer	All patients with active cancer	Alive at hospital discharge	Deceased at hospital discharge	*P-*value
Solid NADC				
Overall (all types pooled)	62	29	33	
Lung	22 (35.5)	11 (37.9)	11 (33.3)	0.09
Intestinal tract	14 (22.6)	4 (13.8)	10 (30.4)	
Upper aerodigestive tract	7 (11.3)	6 (20.7)	1 (3.0)	
Others	19 (30.6)	8 (27.6)	11 (33.3)	
Metastatic	33 (53.2)	14 (48.3)	19 (57.6)	0.81
Stage				
Inaugural admission	30 (48.3)	14 (48.3)	16 (48.5)	0.97
Response	16 (25.8)	8 (27.6)	8 (24.2)	
Relapse	4 (6.5)	2 (6.9)	2 (6.1)	
Refractory	12 (19.4)	5 (17.2)	7 (21.2)	
Haematological NADC				
Overall (all types pooled)	35	24	11	
Hodgkin lymphoma	10 (28.6)	6 (25.0)	4 (36.4)	0.50
Non-AIDS-defining NHL ^a^	11 (31.4)	7 (29.2)	4 (36.4)	
Acute leukaemia	9 (25.7)	8 (33.3)	1 (9.1)	
Myeloma	5 (14.3)	3 (12.5)	2 (18.2)	
Stage				
Inaugural admission	21 (60.0)	15 (62.4)	6 (54.5)	0.48
Response	9 (25.7)	7 (29.2)	2 (18.2)	
Relapse	3 (8.6)	1 (4.2)	2 (18.2)	
Refractory	2 (5.7)	1 (4.2)	1 (9.1)	
Solid ADC				
Overall (all types pooled)	20	15	5	NA
Kaposi sarcoma	19 (95.0)	14 (93.3)	5 (100)	
Cervix cancer	1 (5.0)	1 (6.7)	0	
Haematological ADC				
Overall (all types pooled)	86	59	27	
Primary cerebral NHL	4 (4.6)	3 (5.0)	1 (3.7)	0.04
Burkitt lymphoma	35 (40.7)	29 (49.2)	6 (22.2)	
Other AIDS-defining NHL ^b^	47 (54.7)	27 (45.8)	20 (74.1)	
Stage				
Inaugural admission	65 (75.6)	44 (74.5)	21 (77.8)	0.58
Response	12 (14.0)	10 (17.0)	2 (7.4)	
Relapse	7 (8.1)	4 (6.8)	3 (11.1)	
Refractory	2 (2.3)	1 (1.7)	1 (3.7)	

Variables are exposed as number (percentage)

NADC, non-AIDS-defining cancer; ICU, intensive care unit; NHL, non-Hodgkin lymphoma; ADC, AIDS-defining cancer

^a^Including adult T-cell lymphoma/leukaemia (*n* = 4), T-cell lymphoma (*n* = 4), NK-cell lymphoma (*n* = 1), Waldenström disease (*n* = 1), and marginal zone lymphoma (*n* = 1)

^b^Including diffuse large B cell lymphoma (*n* = 33), plasmablastic lymphoma (*n* = 10), and primary effusion lymphoma (*n* = 4)

### Clinical presentation and organ support in patients with and without cancer

Acute respiratory failure, sepsis and non-toxic coma were the leading reasons for ICU admission, whatever the cancer status (Table 1). The extent of organ failures at admission, as reflected by the SOFA score, did not differ between patients with and without neoplasm. Bacterial sepsis and cancer-related non-infectious complications were the most common definite diagnoses of the ICU stay in patients with NADC and ADC. Notably, NHL-related tumour lysis syndrome accounted for one-fourth of admissions in patients with ADC.

Invasive mechanical ventilation was similarly implemented in patients with and without cancer. Patients with ADC were more often treated with vasopressors and renal replacement therapy than those with NADC or no cancer. Half of patients with ADC received anti-neoplastic chemotherapy in the ICU.

### In-hospital mortality

**Fig. 1:**
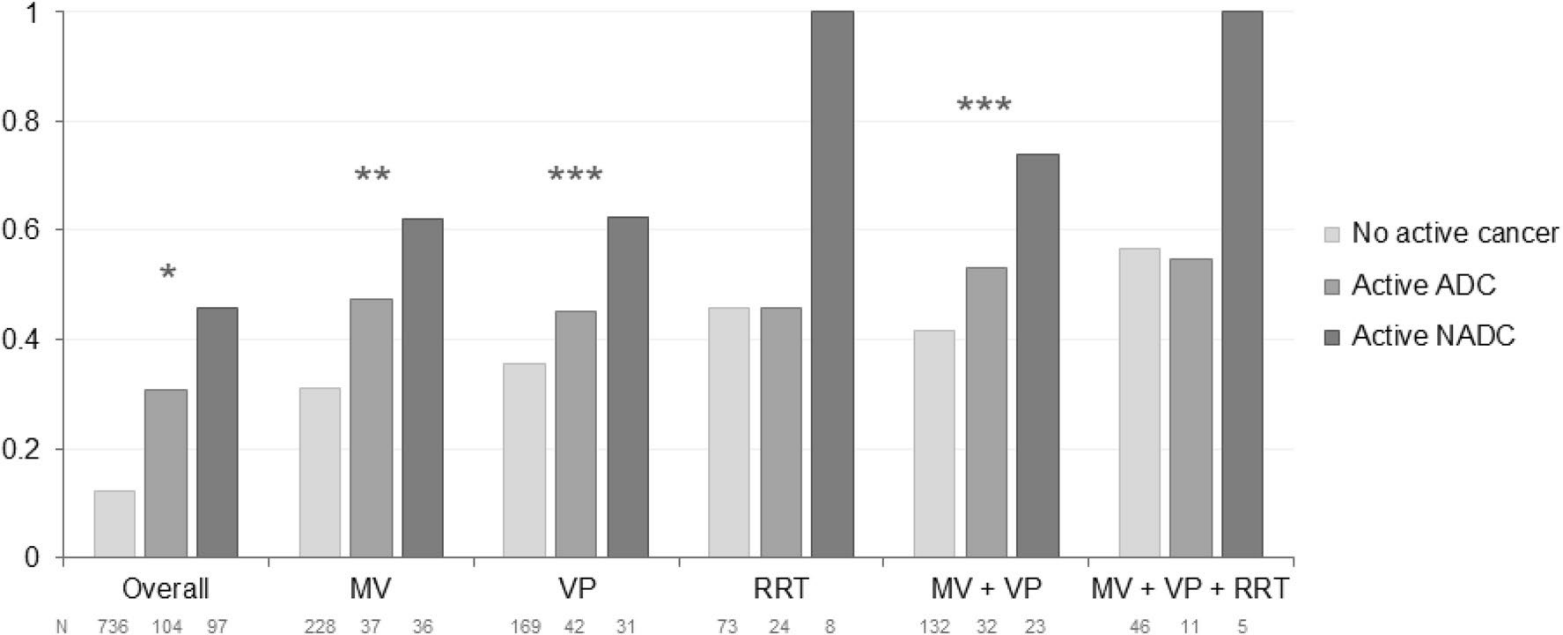
In-hospital mortality according to the cancer status and use of organ support in the ICU. Data are exposed as percentages. MV, mechanical ventilation; VP, vasopressors; RRT, renal replacement therapy; ADC, AIDS-defining cancer; NADC, non-AIDS-defining cancer. *P < 0.0001; **P = 0.005; ***P = 0.01

A total of 167 patients (17.8%) died during their hospital stay (Additional file 1: Table S1). Overall hospital mortality was 12.4% in patients without cancer, 30.2% in patients with ADC and 45.4% in patients with NADC (*P* < 0.0001) (Fig. 1). Around half of deaths in patients with cancer occurred after ICU discharge, which is in the downstream ward, and were related to cancer in most of cases (Table 1). For admissions with bacterial sepsis as the main diagnosis, hospital mortality was 19.4% in patients without cancer, 28.0% in those with ADC and 43.8% in those with NADC (*P* = 0.008).

In the final model, NADC (aOR, 7.00; 95% CI 4.07–12.05; *P* < 0.0001), ADC (aOR, 3.11; 95% CI 1.76–5.51; *P* = 0.0001), liver cirrhosis (aOR, 2.40; 95% CI 1.25–4.60; *P* = 0.008), invasive mechanical ventilation (aOR, 3.45; 95% CI 2.12–5.62; *P* < 0.0001) and the SOFA score value at admission (aOR per point-increase, 1.18; 95% CI 1.12–1.25; *P* < 0.0001) were independently associated with in-hospital death (Table 3, results of the full model in Additional file 1: Table S3). The final model showed a good discriminative ability with an AUROC of 0.86 (95% CI 0.83–0.89) (2000 non-stratified bootstrap replicates).

**Table 3:** Independent predictors of in-hospital death: results of the final model

Variables	aOR	95% CI	*P* value
Active NADC	7.00	4.07–12.05	< 0.0001
Active ADC	3.11	1.76–5.51	0.0001
Invasive mechanical ventilation during the ICU stay	3.45	2.12–5.62	< 0.0001
Liver cirrhosis	2.40	1.25–4.60	0.008
SOFA score value at ICU admission, per 1-point increase	1.18	1.12–1.25	< 0.0001
WHO performance status 3 or 4 (versus ≤ 2)	1.71	0.87–3.35	0.12
WHO performance status 2, 3 or 4 (versus ≤ 1)	1.65	0.99–2.74	0.05
Age, per 10-year increase	1.19	1.00–1.41	0.05
Male sex	0.84	0.53–1.32	0.44

aOR, adjusted odds ratio; CI, confidence interval; NADC, non-AIDS-defining cancer; ICU, intensive care unit; ADC, AIDS-defining cancer; SOFA, sepsis-related organ failure assessment; WHO, World Health Organization

### Treatment limitation decisions

A TLD was pronounced during the ICU stay in 110 patients (11.7%), including organ support withdrawal in 38 (4.0%) and withholding in 72 (7.7%) of them. The prevalence of TLD was 8.0% in patients without cancer, 17.9% in those with ADC and 33.0% in those with NADC (*P* < 0.0001). Death occurred in a context of TLD in 37/69 patients (53.6%) without cancer, 15/32 patients (46.9%) with ADC and 27/44 patients (61.4%) with NADC (*P* = 0.45).

Arguments for TLD are exposed in Table 4. Cancer impacted this decision in 47 patients (92.2%) with ADC or NADC, without significant variation according to the classification or the stage of the disease (Additional file 1: Table S4). Organ failures and non-neoplastic comorbidities were less often considered in patients with cancer than in those without. Only 10 patients (9.1%) in whom a TLD was made had advance directives at ICU admission, a proportion that was not higher in those with neoplasm (Table 4).

**Table 4:** Written arguments for treatment limitation decision in the intensive care unit

Arguments for TLD	Patients with TLD, overall (*n* = 110)	Patients with active cancer (*n* = 51)	Patients without active cancer (*n* = 59)	*P-*value
Organ failures	61 (55.5)	22 (43.1)	39 (76.5)	0.02
Performance status	50 (45.5)	27 (52.9)	23 (45.1)	0.18
Cancer-related	47 (42.7)	47 (92.2)	–	NA
Chronic condition other than cancer	24 (21.8)	6 (11.8)	18 (35.3)	0.02
HIV-related	11 (10.0)	2 (3.9)	9 (17.6)	0.06
Patient’s advance directives	10 (9.1)	6 (11.8)	4 (7.8)	0.51
Requirement for major surgery	6 (5.5)	1 (2.0)	5 (9.8)	0.21
Age	6 (5.5)	2 (3.9)	4 (7.8)	0.68

Data are exposed as number (percentage)

ICU, intensive care unit; TLD, therapeutic limitation decision

### One-year mortality for patients alive at hospital discharge

**Fig. 2:**
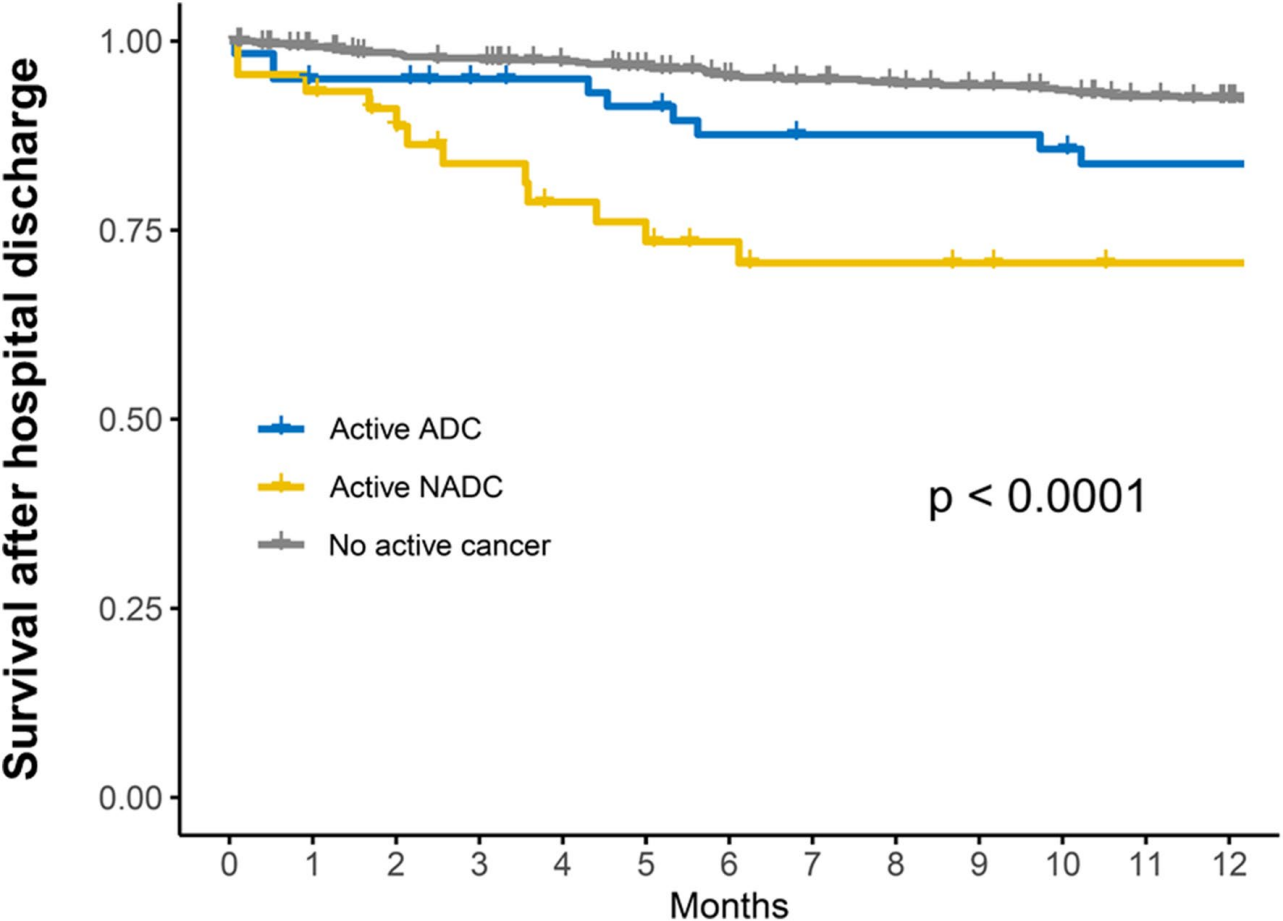
Cumulative survival at one-year according to the cancer status in patients discharged alive from the index hospital admission. Kaplan–Meier curves (with right-censoring at the date of last follow-up information) were compared using the log-rank test. ADC, AIDS-defining cancer; NADC, non-AIDS-defining cancer

At least one follow-up hospital visit was available for 643 (83.3%) out of the 772 patients alive at hospital discharge. Figure 2 shows the cumulative survival at one year in these patients according to the cancer status (right-censoring at the time of last available information), the lowest survival being observed in those with ADC (log-rank test, *P* < 0.0001). Vital status at one year was available for 577 (74.7%) out of these 772 survivors of the index hospital admission (Additional file 1: Table S5): 1 year mortality in these patients was 38/488 (7.8%) in those without cancer, 9/53 (17.0%) in those with ADC (including 5/9 cancer-related deaths) and 12/36 (33.3%) in those with NADC (including 10/12 cancer-related deaths) (*P* < 0.0001).

## Discussion

One-fifth of the 939 critically ill PLHIV included in this multicentre study suffered from an active neoplasm, with a similar prevalence of NADC and ADC. Bacterial sepsis and non-infectious cancer-related events accounted for most of admissions in these patients. Cancer was a predominant argument for TLD and strongly predicted in-hospital death after adjustment on other frailty and severity markers.

The prevalence of NADC was high in our cohort, exceeding rates reported in critically ill PLHIV over the early cART era [9, 18–22]. This trend correlates with evidence that PLVIH, including those with sustained viral control under cART, are at increased hazard for certain infection-related NADC (e.g. EBV-promoted Hodgkin lymphoma) and infection-unrelated NADC (e.g. lung cancer) when compared to age- and sex-matched seronegative individuals [8, 23]. The volume of PLHIV with cancer referred for ICU admission could continue to rise through the coming decade as the burden of NADC is expected to amplify, notably amongst patients aged 65 years or older [4]. Besides, Burkitt lymphoma and diffuse large B cell lymphoma represented most of ADC cases. Those high-grade presentations, which generally prevail in PLHIV [24], are associated with a marked propensity for life-threatening complications entailing ICU admission in patients with NHL, regardless of their HIV status [25, 26]. This may have contributed to the over-representation of ADC in our cohort whilst NADC now distinctly predominate in the overall population of PLHIV [27, 28].

The deleterious effect of cancer on hospital survival has several potential explanations. Most of patients with solid NADC presented with indicators of advanced disease, such as altered performance status, metastatic stage, or admission for a complication directly related to the underlying tumour. The noteworthy prevalence of inaugural admissions may also reflect aggressive phenotypes or late diagnoses. This could be especially relevant for haematological malignancies, with a large subset of patients with tumour lysis syndrome requiring renal replacement therapy or treated with chemotherapy during their ICU stay, as observed in other cohorts [29]. Moreover, patients with cancer were mainly transferred from wards. Early admission from the emergency department, before the worsening of organ failures, has been shown to improve survival in critically ill individuals with neoplasm [30–32]. Next, the additional immune deficiency resulting from cancer and antineoplastic chemotherapies may intrinsically impair survival in PLHIV at the acute phase of critical illnesses. Indeed, amongst patients admitted for bacterial sepsis, in-hospital mortality was greater in those with cancer, as reported in studies not focussed on PLHIV [33, 34]. Lastly, in-hospital fatalities occurring after ICU discharge were far more common in patients with cancer —in this later subgroup, most of deaths occurring in the downstream ward were related to disease progression or new cancer-related complications.

A key question is whether HIV infection impairs per se the outcomes of patients with cancer requiring ICU admission. Data regarding the prognostic impact of HIV infection on the outcome of critically ill patients are conflicting. A large cohort study conducted in the early cART era and including both PLHIV and seronegative controls (matched on age, gender, and medical versus surgical reasons for ICU admission) reported higher unadjusted short-term mortality rates in PLHIV [35]. However, most of studies from the same period found no independent association between HIV infection and survival in patients admitted to the ICU for common conditions such as resuscitated cardiac arrest or the acute respiratory distress syndrome [36, 37]. Also, the severity of HIV-induced immune deficiency, as reflected by the CD4 cell count, does not correlate with short-term outcomes in PLHIV admitted for acute respiratory failure [38], neurological failure [39], or sepsis [40, 41]. Overall, recent case series of critically ill PLHIV showed similar outcomes that those currently reported in seronegative individuals with comparable demographics, comorbidities, and level of organ dysfunctions [11, 21]. Next, to the best of our knowledge, the prognosis of critically ill patients with a given cancer type as never been studied according to the HIV status. Nevertheless, the hospital mortality rates that we observed in our cohort for patients with the most common cancer presentations—namely NHL and lung cancer—were similar to those observed in critically ill seronegative patients with these neoplasms [25, 26, 29, 42, 43]. Altogether, these data suggest that HIV infection has no major impact on the prognosis of critically ill patients with cancer though further studies are warranted to confirm this assumption.

TLD during the ICU stay and in-hospital deaths preceded by such decisions were as frequent in our cohort as in the general population of critically ill patients [44, 45]. Presenting with an active cancer acted as a major determinant for TLD, along with poor baseline health status and the extent of organ failures. HIV infection was only rarely taken in account, contrasting with studies conducted in the early cART era [46, 47]. Overall, these results and the evidence discussed above suggest that, in a context of extended access to cART and improved life expectancy, TLD in PLHIV admitted to the ICU now rests upon a similar making-process than for seronegative individuals, with a substantial impact of cancer prognosis. Strikingly, advance directives were unavailable for most of patients with cancer. Though the frequency of inaugural admissions may partly explain this observation, advance directive completion appears essential for better defining the goals of care in an ageing population of PLHIV in whom the prevalence of malignancies is increasing [48–50].

This work has limitations. First, ADC and NADC were pooled for analyses due to the restricted number of patients with each cancer subtype. Dedicated studies are necessary to appraise the specific prognostic impact of common neoplasms such as lung cancer or AIDS-defining NHL in critically ill PLHIV. Second, owing to the retrospective design, some relevant predictors of outcome might have been omitted, notably the time elapsed from the first symptoms of critical illness to ICU admission. Transient stress in bed availability or reluctance for ICU admission of patients with cancer could have led to delayed referral and impaired prognosis. Along this line, policies for both admission and do-not-resuscitate decisions may differ from one ICU to another, which could affect the external validity of our results. Next, we did not collect information on the course of organ failures throughout the ICU stay. Nevertheless, life-sustaining therapies were entered as active variables in the model for mortality prediction and whether organ failures influenced TLD was extracted from the patient report files. Lastly, as follow-up ended at hospital discharge, we did not explore the effect of critical illness on disease progression, functional outcomes and long-term survival in PLHIV with cancer.

## Conclusions

NADC and ADC are equally prevalent, stand as a leading argument for TLD, and strongly predict in-hospital death in the current population of PLHIV requiring critical care. Enhanced screening procedures for earlier cancer diagnosis, routine policy for prompt ICU admission and promotion of advance directive completion in case of documented neoplasm may constitute important axes for improving the quality of care in these patients.

### Supplementary Information

**Additional file 1.** Recruitment and characteristics of participating intensive care units. **Table S1.** Characteristics of the study population according to the vital status at hospital discharge. **Table S2.** Bacterial sepsis as the main diagnosis in the intensive care unit. **Table S3.** Independent predictors of in-hospital death: full results of the initial model. **Table S4.** Written arguments for treatment limitation decision in patients with cancer. **Table S5.** One-year mortality in patients discharged alive from the index hospital admission. **Figure S1.** Study flowchart.

## Data Availability

The dataset used and analysed in this study is available from the corresponding author on reasonable request.

## Abbreviation

HIV: Human immunodeficiency virus
PLHIV: People living with HIV
cART: Combination antiretroviral therapy
AIDS: Acquired immune deficiency syndrome
ADC: AIDS-defining cancer
NHL: Non-Hodgkin lymphoma
NADC: Non-AIDS-defining cancers
TLD: Treatment limitation decision
ICU: Intensive care unit
STROBE: Strengthening the reporting of observational studies in epidemiology
ABE: Augmented backward elimination
SOFA: Sepsis-related organ failure assessment
SAPS 2: Simplified acute physiology score 2
aOR: Adjusted odd ratio
CI: Confidence interval
AUROC: Receiver operating characteristics curve
EBV: Epstein–Barr virus
