# Correction: Clinical spectrum and prognostic impact of cancer in critically ill patients with HIV: a multicentre cohort study

**DOI:** 10.1186/s13613-024-01254-w

**Published:** 2024-02-14

**Authors:** Piotr Szychowiak, Thierry Boulain, Jean-François Timsit, Alexandre Elabbadi, Laurent Argaud, Stephan Ehrmann, Nahema Issa, Emmanuel Canet, Frédéric Martino, Fabrice Bruneel, Jean-Pierre Quenot, Florent Wallet, Élie Azoulay, François Barbier

**Affiliations:** 1 Médecine Intensive Réanimation, Centre Hospitalier Régional d’Orléans, 14, Avenue de L’Hôpital, 45100 Orléans, France; 2 Réanimation Médicale et des Maladies Infectieuses, Centre Hospitalier Universitaire Bichat-Claude Bernard, Assistance Publique-Hôpitaux de Paris, Paris, France; 3 Médecine Intensive Réanimation, Centre Hospitalier Universitaire Tenon, Assistance Publique-Hôpitaux de Paris, Paris, France; 4 Médecine Intensive Réanimation, Centre Hospitalier Universitaire Edouard Herriot, Hospices Civils de Lyon, Lyon, France; 5 Médecine Intensive Réanimation, Centre Hospitalier Universitaire de Tours, Tours, France; 6 Médecine Intensive Réanimation, Centre Hospitalier Universitaire de Bordeaux, Bordeaux, France; 7 Médecine Intensive Réanimation, Centre Hospitalier Universitaire de Nantes, Nantes, France; 8 Médecine Intensive Réanimation, Centre Hospitalier Universitaire de La Guadeloupe, Pointe-À-Pitre, France; 9 Réanimation et Unité de Surveillance Continue, Centre Hospitalier de Versailles, Le Chesnay, France; 10 Médecine Intensive Réanimation, Centre Hospitalier Universitaire de Dijon-Bourgogne, Dijon, France; 11 Médecine Intensive Réanimation, Centre Hospitalier Universitaire Lyon Sud, Hospices Civils de Lyon, Lyon, France; 12 Médecine Intensive Réanimation, Centre Hospitalier Universitaire Saint-Louis, Assistance Publique-Hôpitaux de Paris, Paris, France

**Correction: Annals of Intensive Care (2023) 13:74** 10.1186/s13613-023-01171-4

In the original publication of the article, the authors found the below errors.

The fourth author name Alexandre Elabbadi was incorrectly written as Alexandre Elabaddi.

The legend inside the Fig. 2 is reversed between the NADC and ADC groups. The corrected Fig. 2 is given in this correction article.

**Fig. 2:**
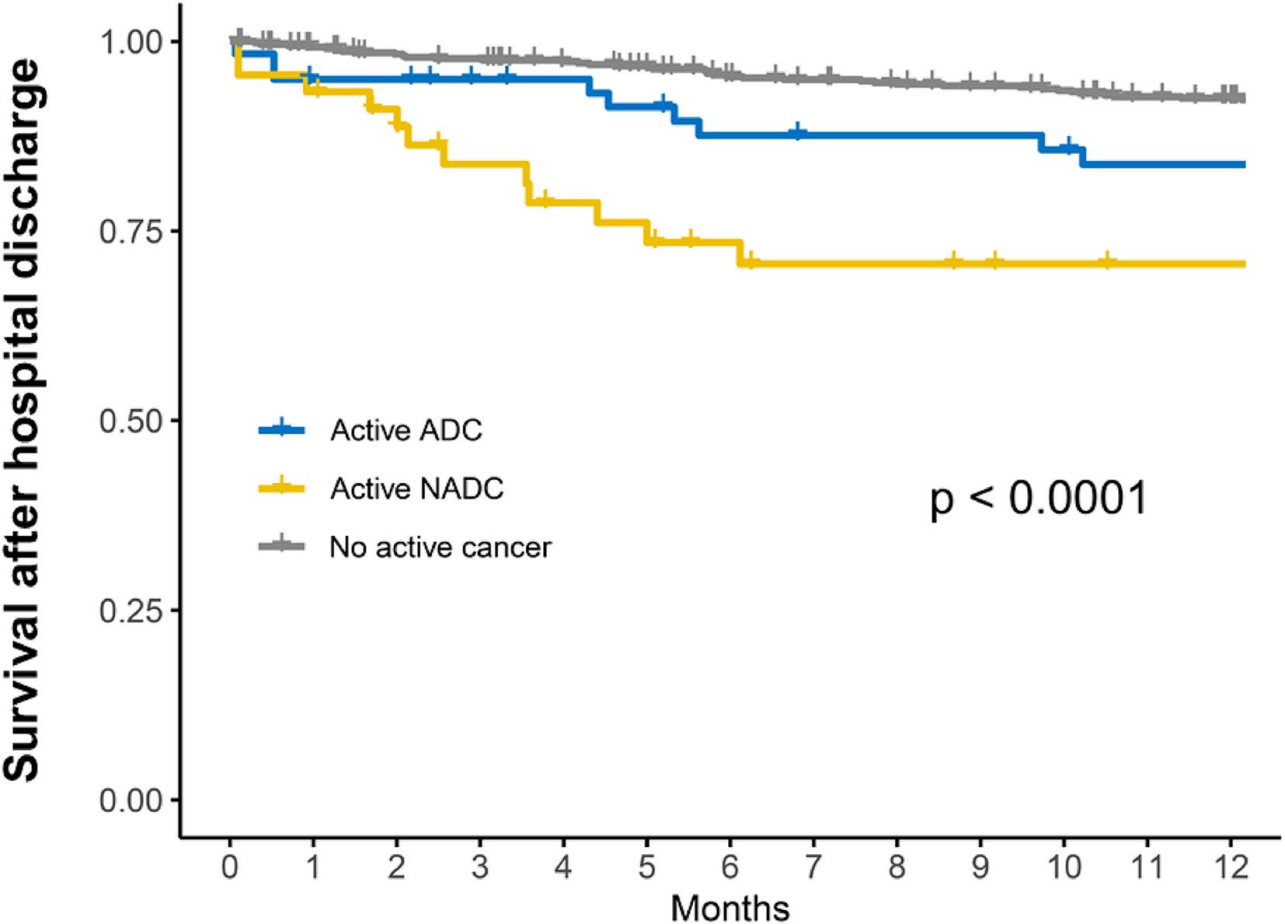
Cumulative survival at one-year according to the cancer status in patients discharged alive from the index hospital admission. Kaplan–Meier curves (with right-censoring at the date of last follow-up information) were compared using the log-rank test. ADC, AIDS-defning cancer; NADC, non-AIDS-defning cancer

The original article [1] has been corrected.
