# Supplementary material for: Clinical spectrum and prognostic impact of cancer in critically ill patients with HIV: a multicentre cohort study
Source: Ann Intensive Care. 2023 Aug 22;13:74. doi: 10.1186/s13613-023-01171-4 (PMC10444715; doi:10.1186/s13613-023-01171-4)
Supplement: Supplementary file 1 — Additional file 1. Recruitment and characteristics of participating intensive care units. Table S1. Characteristics of the study population according to the vital status at hospital discharge. Table S2. Bacterial sepsis as the main diagnosis in the intensive care unit. Table S3. Independent predictors of in-hospital death: full results of the initial model. Table S4. Written arguments for treatment limitation decision in patients with cancer. Table S5. One-year mortality in patients discharged alive from the index hospital admission. Figure S1. Study flowchart. [file 13613_2023_1171_MOESM1_ESM.docx]

**ELECTRONIC SUPPLEMENT**

**Clinical spectrum and prognostic impact of cancer in critically ill patients with HIV: a multicentre cohort study**

Piotr Szychowiak, MD, MSc; Thierry Boulain, MD; Jean-François Timsit, MD, PhD; Alexandre Elabaddi, MD; Laurent Argaud, MD, PhD; Stephan Ehrmann, MD, PhD; Nahema Issa, MD; Emmanuel Canet, MD, PhD; Frédéric Martino, MD; Fabrice Bruneel, MD; Jean-Pierre Quenot, MD, PhD; Florent Wallet, MD; Élie Azoulay, MD, PhD; François Barbier, MD, PhD

**Recruitment and characteristics of participating intensive care units**

All the intensive care units (ICU) contributing to the GRRROH (*Groupe de Recherche Respiratoire en Réanimation Onco-Hématologique*, [www.grrroh.fr](http://www.grrroh.fr)) research network were solicited for voluntary participation to the study. This network has an internationally recognized expertise in the conduct of observational and interventional studies investigating the clinical presentation, management, and outcome of all populations of immunocompromised patients admitted to the ICU, notably those with cancer or HIV infection. The GRRROH network currently gathers 32 medical ICUs in France, including 24 ICUs located in university hospitals (total number of medical ICUs located in university hospitals in France, n = 52), 25 ICUs with an oncological and/or onco-haematological unit in the same hospital, and 30 ICUs with an infectious disease unit in the same hospital. Among the 12 GRRROH-affiliated ICUs which participated to the study, 3 also contribute to CRICS-TRIGGERSEP (Clinical Research in Intensive Care and Sepsis - Trial Group for Global Evaluation and Research in Sepsis, [www.crics-triggersep.org](http://www.crics-triggersep.org)) network for research in intensive care. These 12 participating ICUs manage a high volume of immunocompromised hosts as part of their routine patient recruitment. Criteria for ICU admission are not standardized and depend on local policies. The decision-making process leading to treatment limitations strictly complies with French laws on end-of-life practices but may vary from one ICU to another.

**Table S1.** Characteristics of the study population according to the vital status at hospital discharge

|  | **All patients**  (n = 939) | **Alive at hospital discharge**  (n = 772) | **Deceased at hospital discharge**  (n = 167) | ***P*-value** |
| --- | --- | --- | --- | --- |
| **Male sex** | 670 (71.3) | 551 (71.4) | 119 (71.3) | 1 |
| **Age, years** | 52 (43-59) | 51 (42-59) | 55 (48-63) | <0.0001 |
| **Body mass index, kg.m^-2^** ^a^ | 22.8 (10.9-55.4) | 22.8 (12.2- 43.6) | 23.4 (10.9-55.4) | 0.39 |
| **Precarity** ^b^ | 136 (14.5) | 111 (14.4) | 25 (15.0) | 0.81 |
| **Living in a nursing home or LTCF** | 47 (5.0) | 41 (5.3) | 6 (3.6) | 0.47 |
| **WHO performance status**  0  1-2  3-4 | 514 (54.7)  334 (35.6)  91 (9.7) | 448 (58.0)  263 (34.1)  61 (7.9) | 66 (39.5)  71 (42.5)  30 (18.0) | <0.0001 |
| **HIV-related characteristics**  Newly diagnosed HIV infection ^c^  CD4 cell count at admission, per µL ^d^  HIV viral load at admission, per µL ^e^  Non-inaugural admission  cART at admission  Baseline CD4 cell count, per µL ^f^  Baseline HIV viral load, per µL ^g^  History of AIDS-defining OI  History of HIV encephalitis  History of Castleman disease  History of ADC (remission or cured)  NHL  Kaposi sarcoma  Cervix cancer | 127 (13.5)  51 (20-147)  5.10^5^ (10^5^-10^6^)  812 (86.5)  699 (74.4)  370 (180-600)  <50 (<50-<50)  301 (32.1)  21 (2.2)  16 (1.7)  50 (5.3)  11 (1.2)  39 (4.2)  1 (0.1) | 105 (13.6)  55 (20-158)  5.10^5^ (10^5^-10^6^)  667 (86.4)  572 (74.1)  392 (198-603)  <50 (<50-75)  240 (31.1)  17 (2.2)  13 (1.7)  40 (5.2)  7 (0.9)  32 (4.1)  1 (0.1) | 22 (13.2)  34 (22-123)  10^6^ (10^5^-10^6^)  145 (86.8)  127 (76.0)  280 (122-500)  <50 (<50-50)  61 (36.5)  4 (2.4)  3 (1.8)  10 (6.0)  4 (2.4)  7 (4.2)  0 | 1  0.57  0.55  1  0.63  0.005  0.25  0.17  1  1  0.67  0.11  0.98  1 |
| **Non-neoplastic chronic conditions**  Tobacco use (current or past)  Chronic alcohol abuse (current or past)  Drug addiction (current or past)  IVDU  Diabetes mellitus  Dyslipidemia  Hypertension  Respiratory  COPD  Sleep apnea  Chronic respiratory failure  Cardiac  CAD  Chronic cardiac diseases other than CAD  Congestive heart failure  Peripheral artery disease  Chronic kidney disease  Chronic dialysis  Kidney transplantation  Hepatic  Chronic HBV infection  Chronic HCV infection  Liver cirrhosis  Neurological  Cerebrovascular disease  Cognitive disorders | 305 (32.5)  195 (20.8)  180 (19.2)  72 (7.7)  134 (14.3)  94 (10.0)  231 (24.6)  199 (21.2)  107 (11.4)  21 (2.2)  23 (2.4)  175 (18.6)  92 (9.8)  105 (11.2)  26 (2.8)  37 (3.9)  171 (18.2)  30 (3.2)  21 (2.2)  200 (21.3)  72 (7.7)  111 (11.8)  74 (7.9)  105 (11.2)  38 (4.0)  18 (1.9) | 266 (34.5)  167 (21.6)  157 (20.3)  62 (8.0)  105 (13.6)  75 (9.7)  192 (24.9)  171 (22.2)  89 (11.5)  21 (2.7)  18 (2.3)  143 (18.5)  76 (9.8)  85 (11.0)  22 (2.8)  34 (4.4)  136 (17.6)  24 (3.1)  17 (2.2)  161 (20.9)  58 (7.5)  89 (11.5)  50 (6.5)  90 (11.7)  29 (3.8)  15 (1.9) | 39 (23.4)  28 (16.8)  23 (13.8)  10 (6.0)  29 (17.4)  19 (11.4)  39 (23.4)  28 (16.8)  18 (10.8)  0 (0.0)  5 (3.0)  32 (19.2)  16 (9.6)  20 (12.0)  4 (2.4)  3 (1.8)  35 (21.0)  6 (3.6)  4 (2.4)  39 (23.4)  14 (8.4)  22 (13.2)  24 (14.4)  15 (9.0)  9 (5.4)  3 (1.8) | 0.007  0.19  0.06  0.43  0.25  0.61  0.75  0.14  0.89  0.06  0.82  0.83  1  0.82  0.95  0.18  0.37  0.94  0.78  0.47  0.82  0.64  0.001  0.42  0.45  1 |
| **Solid organ transplantation** | 24 (2.6) | 20 (2.6) | 4 (2.4) | 1 |

**Table S1 (continued).**

|  | **All patients**  (n = 939) | **Alive at hospital discharge**  (n = 772) | **Deceased at hospital discharge**  (n = 167) | ***P*-value** |
| --- | --- | --- | --- | --- |
| **History of NADC (remission or cured)**  Any  Solid NADC  Haematological NADC | 53 (5.6)  41 (5.5)  13 (1.4) | 43 (5.6)  34 (4.4)  10 (1.3) | 10 (6.0)  7 (4.2)  3 (1.8) | 0.83  0.90  0.71 |
| **Active NADC** ^h^  Any  Solid  Haematological | 97 (7.1)  62 (6.6)  35 (3.7) | 53 (6.9)  29 (3.7)  24 (3.1) | 44 (26.3)  33 (19.8)  11 (5.6) | <0.0001  <0.0001  0.04 |
| **Active ADC** ^h^  Any  NHL  Kaposi sarcoma  Cervix cancer | 106 (11.7)  86 (9.2)  19 (2.0)  1 (0.1) | 74 (9.6)  59 (7.6)  14 (1.8)  1 (0.1) | 32 (19.2)  27 (16.2)  5 (3.0)  0 | 0.001  0.001  0.36  1 |
| **McCabe score**  0  1  2 | 695 (74.0)  189 (20.1)  55 (5.9) | 603 (78.1)  137 (17.7)  32 (4.1) | 92 (55.1)  52 (31.1)  23 (13.8) | <0.0001 |
| **Type of ICU admission**  Medical  Unscheduled surgery  Scheduled surgery | 904 (96.3)  13 (1.4)  22 (2.3) | 743 (96.2)  11 (1.4)  18 (2.3) | 161 (96.4)  2 (1.2)  4 (2.4) | 0.97 |
| **Direct ICU admission**  Direct ^i^  Transfer from wards | 603 (64.2)  336 (35.8) | 529 (68.5)  243 (31.5) | 74 (44.3)  93 (55.7) | <0.0001 |
| **SAPS 2 at ICU admission** | 36 (26-51) | 34 (24-46) | 51 (39-69) | <0.0001 |
| **SOFA score at ICU admission** | 4 (2-6) | 3 (1-5) | 7 (4-12) | <0.0001 |
| **Main reason for ICU admission**  Acute respiratory failure  Sepsis / septic shock  Coma (non-toxic)  Acute kidney failure  Drug overdose  Metabolic  Shock (other than septic)  Cardiac arrest  Others | 320 (34.1)  172 (18.3)  165 (17.6)  56 (6.0)  47 (5.0)  47 (5.0)  28 (3.0)  16 (1.7)  88 (9.3) | 265 (34.3)  129 (16.7)  142 (18.4)  48 (6.2)  47 (6.1)  38 (4.9)  18 (2.3)  4 (0.5)  81 (10.6) | 55 (32.9)  43 (25.7)  23 (13.8)  8 (4.8)  0  9 (5.4)  10 (6.0)  12 (7.2)  7 (4.2) | <0.0001 |
| **Neutropenia at ICU admission** | 46 (4.9) | 31 (4.0) | 15 (9.0) | 0.02 |
| **Main diagnosis of the ICU stay**  Cancer-related non-infectious complication  NADC  ADC  Tumor lysis syndrome  Non-infectious complication of chronic condition  Bacterial sepsis  Non-bacterial non-AIDS-defining infection  Malaria  COVID-19  Influenza  Others  AIDS-defining OI  *Pneumocystis jirovecii* pneumonia  Cerebral toxoplasmosis  Tuberculosis  Other OI  cART-related toxicity  Miscellaneous | 116 (12.4)  47 (5.0)  69 (7.7)  27 (3.1)  242 (25.8)  263 (28.0)  43 (4.6)  20 (2.1)  7 (0.7)  7 (0.7)  9 (1.0)  156 (16.6)  69 (7.3)  30 (3.2)  14 (1.5)  43 (4.6)  7 (0.7)  112 (11.9) | 74 (9.6)  27 (3.5)  47 (6.1)  19 (2.5)  222 (28.8)  202 (26.2)  39 (5.1)  19 (2.5)  6 (0.8)  5 (0.6)  9 (1.1)  136 (17.6)  60 (7.8)  26 (3.4)  12 (1.6)  38 (4.9)  6 (0.8)  93 (12.0) | 42 (25.1)  20 (12.0)  22 (13.2)  8 (4.8)  20 (12.0)  61 (36.5)  4 (2.4)  1 (0.6)  1 (0.6)  2 (1.2)  0  20 (12.0)  9 (5.4)  4 (2.4)  2 (1.2)  5 (3.0)  1 (0.6)  19 (11.4) | <0.0001  <0.0001  0.0015  0.12  <0.0001  0.008  0.16  0.30  0.08  0.63  1  0.80 |

**Table S1 (continued).**

|  | **All patients**  (n = 939) | **Alive at hospital discharge**  (n = 772) | **Deceased at hospital discharge**  (n = 167) | ***P*-value** |
| --- | --- | --- | --- | --- |
| **Organ support in the ICU**  High-flow nasal oxygen therapy  Non-invasive ventilation  Invasive mechanical ventilation  Vasopressors  Renal replacement therapy for AKI  VA-ECMO  VV-ECMO | 96 (10.2)  73 (7.8)  301 (32.1)  242 (25.8)  105 (11.2)  4 (0.4)  10 (1.1) | 72 (9.3)  59 (7.6)  191 (24.7)  144 (18.7)  61 (7.9)  2 (0.3)  3 (0.4) | 24 (14.4)  14 (8.4)  110 (65.9)  98 (58.7)  44 (26.3)  2 (1.2)  7 (4.2) | 0.07  0.75  <0.0001  <0.0001  <0.0001  0.15  0.0004 |
| **Chemotherapy in the ICU** | 71 (7.6) | 46 (6.0) | 25 (15.0) | 0.0003 |
| **Adverse events during the ICU stay**  Ventilator-associated pneumonia  VAPA  Infection or colonization with MDRB  *Clostridioides difficile* infection  ARDS  Cardiac arrest | 54 (5.8)  4 (0.4)  121 (12.9)  13 (1.4)  124 (13.2)  31 (3.3) | 27 (3.5)  1 (0.1)  92 (11.9)  10 (1.3)  58 (7.5)  7 (0.9) | 27 (16.2)  3 (1.8)  29 (17.4)  3 (1.8)  66 (39.5)  24 (14.4) | <0.0001  0.02  0.001  0.89  <0.0001  <0.0001 |
| **TLD during the ICU stay**  Organ support withdrawal  Organ support withholding | 110 (11.7)  38 (4.0)  72 (7.7) | 27 (3.5)  0  27 (3.5) | 83 (49.7)  38 (22.8)  45 (26.9) | <0.0001  <0.0001  <0.0001 |
| **ICU readmission** | 48 (5.3) | 37 (5.0) | 11 (6.6) | 0.53 |
| **Outcomes**  ICU length of stay, days  Hospital length of stay, days  In-ICU death | 5 (3-9)  19 (10-36)  112 (11.9) | 5 (3-8)  18 (10-35)  - | 7 (3-16)  20 (10-40)  112 (67.1) | <0.0001  0.52  NA |

*Table S1 footnote*

Data are exposed as number (percentage) or median (interquartile range).

LCTF, long-term care facility; WHO, World Health Organization; cART, combination antiretroviral therapy; OI, opportunistic infection; ADC, AIDS-defining cancer; NHL, non-Hodgkin lymphoma; IVDU, intravenous drug use; COPD, chronic obstructive pulmonary disease; CAD, coronary heart disease; NADC, non-AIDS-defining cancer; ICU, intensive care unit; SAPS 2, simplified acute physiology score 2; SOFA, sepsis-related organ failure assessment; OI, opportunistic infection; AKI, acute kidney injury; VA/VV-ECMO, veino-arterial/veino-veinous extracorporeal membrane oxygenation; VAPA, ventilator-associated pulmonary aspergillosis; MDRB, multidrug-resistant bacteria; ARDS, acute respiratory distress syndrome

^a^ Missing value for 317/939 patients; ^b^ Homeless (n = 29), migrants (n = 54), incarcerated patient (n = 1) and other complex social situations (n = 52); ^c^ Diagnosis of HIV infection during the same hospital stay (inaugural admission); ^d^ Missing value for 16/127 patients with newly diagnosed HIV infection;

^e^ Missing value for 22/127 patients with newly diagnosed HIV infection; ^f^ Within 6 months prior to ICU admission, missing values for 273/812 patients with previously known HIV infection; ^g^ Within 6 months prior to ICU admission, missing values for 196/812 patients with previously known HIV infection; ^h^ Solid or haematological cancer not in remission – that is, either inaugural admission, response to first-line therapy, relapse, or refractory disease; ^i^ ICU admission within the first 24 hours following hospital admission

**Table S2.** Bacterial sepsis as the main diagnosis in the intensive care unit

|  | **Patients with bacterial sepsis**  (n = 263) |
| --- | --- |
| **Infection sites**  Pulmonary  Urinary tract  Intra-abdominal  Central nervous system  Skin and soft tissue  Catheter-related bloodstream infection  Endocarditis  Primary bloodstream infection  Others | 127 (48.3)  29 (11.0)  30 (11.4)  13 (4.9)  13 (4.9)  9 (3.4)  10 (3.8)  13 (4.9)  19 (7.2) |
| **Pathogens**  Enterobacterales  *Pseudomonas aeruginosa*  Other non-fermenting Gram-negative bacteria  *Haemophilus spp.*  *Staphylococcus aureus*  Coagulase-negative staphylococci  *Streptococcus pneumoniae*  Other *Streptococcus spp.*  *Enterococcus spp.*  *Legionella pneumophila*  *Mycoplasma pneumoniae*  Anaerobes  *Clostridioides difficile*  No documentation | 68 (25.9)  15 (5.7)  2 (0.8)  10 (3.8)  27 (10.3)  3 (1.1)  37 (14.1)  9 (3.4)  8 (3.0)  2 (0.8)  1 (0.4)  6 (2.3)  1 (0.4)  95 (36.1) |

*Table S2 footnote*

Data are exposed as number (percentage).

**Table S3.** Independent predictors of in-hospital death: full results of the initial model

| **Variables** | **Inclusion frequency** | **OR median** | **CI lower** | **CI upper** | **RMSD ratio** | **RCB** |
| --- | --- | --- | --- | --- | --- | --- |
| Active NADC ^a^ | 1 | 8.55 | 4.66 | 16.51 | 1.13 | 3.02 |
| Invasive mechanical ventilation | 1 | 3.84 | 2.12 | 7.28 | 1.18 | 1.94 |
| Active ADC ^a^ | 0.99 | 3.25 | 1.63 | 6.59 | 1.16 | 3.85 |
| Liver cirrhosis | 0.97 | 2.82 | 1 | 5.66 | 1.12 | 6.63 |
| WHO performance status 3 or 4 (versus ≤2) | 1 | 1.76 | 0.76 | 4.14 | 1.25 | 8.80 |
| WHO performance status 2, 3 or 4 (versus ≤1) | 0.81 | 1.68 | 1 | 2.99 | 1.27 | 13.09 |
| AIDS-related OI ^b^ | 0.53 | 1.34 | 0.68 | 2.57 | 1.06 | 65.55 |
| Septic shock ^b^ | 0.55 | 1.32 | 1 | 2.24 | 1.07 | 67.70 |
| SOFA score value, per 1-point increase ^c^ | 1 | 1.17 | 1.09 | 1.28 | 1.05 | 3.64 |
| Age, per 10-year increase | 1 | 1.02 | 0.99 | 1.04 | 1.16 | 6.38 |
| Tobacco use | 0.50 | 1.00 | 0.48 | 1.35 | 1.06 | 101.6 |
| SAPS 2 value, per 1-point increase ^c^ | 1 | 1.00 | 0.99 | 1.02 | 1.11 | 15.37 |
| Diabetes mellitus | 0.46 | 1 | 0.67 | 2.33 | 1.08 | 94.56 |
| Male sex | 1 | 0.92 | 0.54 | 1.55 | 1.12 | 24.08 |
| Drug addiction | 0.57 | 0.70 | 0.34 | 1 | 1.14 | 73.42 |
| Peripheral arterial disease | 0.99 | 0.18 | 7.80E-08 | 0.53 | 4.54 | 46.91 |

*Table S3 footnote*

OR, odds ratio; CI, confidence interval; RMSD, root mean square difference; RCB, relative conditional bias; NADC, non-AIDS-defining cancer; ADC, AIDS-defining cancer; WHO, World Health Organization; OI, opportunistic infection; SOFA, sepsis-related organ failure assessment.

^a^ Active cancer is defined as solid or haematological neoplasm with either inaugural admission, response to first-line therapy, relapse, or refractory disease; ^b^; Primary diagnosis of the intensive care unit (ICU) stay; ^c^ At ICU admission

**Table S4.** Written arguments for treatment limitation decision in patients with cancer

| **Arguments for TLD** | **Patients with cancer, overall**  n = 51 | **Patients with NADC**  n = 32 | **Patients with ADC**  n = 19 | **Patients with inaugural admission** ^a^  n = 25 | **Patients with non-inaugural admission**  n = 26 |
| --- | --- | --- | --- | --- | --- |
| Organ failures | 22 (43.1) | 12 (37.5) | 10 (52.6) | 13 (52.0) | 9 (34.6) |
| Performance status | 27 (52.9) | 18 (56.3) | 9 (47.4) | 12 (48.0) | 15 (57.7) |
| Cancer-related | 47 (92.2) | 30 (93.8) | 17 (89.5) | 25 (100) | 22 (84.6) |
| Chronic condition other than cancer | 6 (11.8) | 5 (15.6) | 1 (5.3) | 4 (16.0) | 2 (7.7) |
| HIV-related | 2 (3.9) | 0 | 2 (10.5) | 0 | 2 (7.7) |
| Patient’s advance directives | 6 (11.8) | 1 (3.1) ^b^ | 5 (26.3) ^b^ | 3 (12.0) | 3 (11.5) |
| Requirement for major surgery | 1 (2.0) | 1 (3.1) | 0 | 0 | 1 (3.8) |
| Age | 2 (3.9) | 2 (6.3) | 0 | 1 (4.0) | 1 (3.8) |

*Table S4 footnote*

Data are exposed as number (percentage).

TLD, treatment limitation decision; ADC/NADC, AIDS-defining/non-AIDS-defining cancer

^a^ Diagnosis of cancer during the index hospital stay

^b^ *P* = 0.02 (*P* >0.05 for all other comparisons)

**Table S5.** One-year mortality in patients discharged alive from the index hospital admission

| **One-year vital status** | **All patients alive at hospital discharge**  (n = 772) | **Patients without active cancer**  (n = 645) | **Patients with active ADC**  (n = 74) | **Patients with active NADC**  (n = 53) | ***P*-value** |
| --- | --- | --- | --- | --- | --- |
| Available information | 577/772 (74.7) | 488/645 (75.7) | 53/74 (71.6) | 36/53 (67.9) | 0.37 |
| **One-year mortality, overall**  Cancer-related deaths | 59/577 (10.2)  15/59 (25.4) | 38/488 (7.8)  - | 9/53 (17.0) ^a^  5/9 (55.6) | 12/36 (33.3) ^b^  10/12 (83.3) | <0.0001  - |

*Table S5 footnote*

Variables are exposed as number (percentage).

ADC, AIDS-defining cancer; NADC, non-AIDS-defining cancer

^a^ One-year mortality was 9/17 (52.9%) in patients with solid NADC (including 7/9 cancer-related deaths) and 3/19 (15.8%) in those with haematological NADC (including 3/3 cancer-related deaths) (*P* = 0.03)

^b^ One-year mortality was 6/42 (14.3%) in patients with AIDS-related lymphoma (including 4/6 cancer-related deaths), 2/10 (20.0%) in those with Kaposi sarcoma (both AIDS-related) and 1/1 for AIDS-related cervix cancer (*P* = 0.07)

**Figure S1.** Study flowchart

Exclusion due to missing essential data:

cART use at ICU admission, n = 3

Vital status at hospital discharge, n = 10

**939 patients included in the study population**

952 patients with HIV with a first admission in the 12 participating ICUs over the inclusion period (January 2015 – June 2020)

772 patients (82.2%) alive at hospital discharge

167 patients (11.8%) deceased at hospital discharge

*Figure S1 footnote*

cART, combination antiretroviral therapy; ICU, intensive care unit
